# Integrating Multi–Omics Data for Gene-Environment Interactions

**DOI:** 10.3390/biotech10010003

**Published:** 2021-01-29

**Authors:** Yinhao Du, Kun Fan, Xi Lu, Cen Wu

**Affiliations:** Department of Statistics, Kansas State University, Manhattan, KS 66506, USA; ydu@ksu.edu (Y.D.); kfan@ksu.edu (K.F.); xilu@ksu.edu (X.L.)

**Keywords:** Gene-environment (G×E) interactions, integrated analysis, multidimensional data, high-dimensional variable selection

## Abstract

Gene-environment (G×E) interaction is critical for understanding the genetic basis of complex disease beyond genetic and environment main effects. In addition to existing tools for interaction studies, penalized variable selection emerges as a promising alternative for dissecting G×E interactions. Despite the success, variable selection is limited in terms of accounting for multidimensional measurements. Published variable selection methods cannot accommodate structured sparsity in the framework of integrating multiomics data for disease outcomes. In this paper, we have developed a novel variable selection method in order to integrate multi-omics measurements in G×E interaction studies. Extensive studies have already revealed that analyzing omics data across multi-platforms is not only sensible biologically, but also resulting in improved identification and prediction performance. Our integrative model can efficiently pinpoint important regulators of gene expressions through sparse dimensionality reduction, and link the disease outcomes to multiple effects in the integrative G×E studies through accommodating a sparse bi-level structure. The simulation studies show the integrative model leads to better identification of G×E interactions and regulators than alternative methods. In two G×E lung cancer studies with high dimensional multi-omics data, the integrative model leads to an improved prediction and findings with important biological implications.

## 1. Introduction

Gene-environment interactions reveal how the changes in environmental exposures mediate the contribution of genetic factors in order to influence the variations in disease traits, which makes it critical in understanding the comprehensive genetic architecture of complex diseases [1,2]. Traditionally, G×E interaction studies have mainly been conducted within the framework of genetic association studies in order to hunt down the important main and interaction effects that are associated with the disease phenotypes [3,4].

Most of the existing G×E studies are one-dimensional, in that the interactions between environmental factors and one type of genetic factor (such as gene expression or SNPs) have been considered. In the multi-omics era, there is a pressing need to account for multi-platform measurements in G×E studies. Consider a G×E analysis with environmental factors and gene expression (GE) as the G factors. In addition, DNA methylation (DM) and copy number alterations (CNA), which are the regulators of the genetic factors, are also available. A typical G×E analysis only focuses on the interaction effects that involve the G factor (GE) and ignores its regulators, losing the extra power of elucidating the genetic basis of complex disease while using multi-level omics data.

Integrating multi-omics data for prognostic outcomes has mainly been conducted using parallel and horizontal integration strategies [5]. With the parallel integration, different types of omics measurements are treated equally, and important associations between these measurements and the prognostic outcome are identified in a joint model. On the other hand, the hierarchical integration fully accounts for the regulatory information by accommodating the indirect effects of regulators, such as DM and CNA, on the prognostic outcomes that are mediated through GEs. Meanwhile, the direct effects of regulators on the cancer outcomes, which have not been captured by GEs through other mechanisms, such as post-transcriptional regulations, should also been taken into consideration.

Given the availability of multi-omics features, the major limitation of existing G×E interaction studies lies in the incapability of integrating regulators in the interaction model under prognostic outcomes, which has motivated us to develop a two stage integrative model for G×E interaction analysis while using multi-level cancer omics data. At the first stage, the sparse regulatory relationship has been determined through penalization, where the linear regulatory modelling [6], or LRM, has been adopted in order to identify the sets of regulators that influence the sets of GEs, as well as the residuals of gene expression and residuals of regulators that cannot be captured by the LRMs. At the second stage, the LRMs and both types of residuals are treated as direct effects on cancer outcomes in the G×E model, and penalization has been conducted in order to identify the important main and interaction effects.

In the past decade, the effectiveness of regularization for G×E interaction studies has been increasingly witnessed [7]. Extension of the technique for an integrated interaction study is not trivial. Our method significantly advances from existing integration studies not tailored for interaction structures and interaction analysis ignoring the multidimensional omics measurements. Extensive simulation studies, have been performed to demonstrate the advantage of the proposed method over multiple alternatives. Our method leads to main and interaction effects with sensible biological implications and improved prediction performance in two case studies of the lung cancer data (LUSC and LUAD) from TCGA.

## 2. Method

Let $Y_{n\times 1}$ denote cancer outcome, $E_{n\times q}=\left(E_{1},\cdots ,E_{q}\right)$ denote the *q* environmental factors, $G_{n\times p_{g}}=\left(G_{1},\cdots ,G_{p_{g}}\right)$ denote the $p_{g}$ gene expressions, and $R_{n\times p_{r}}=\left(R_{1},\cdots ,R_{p_{r}}\right)$ denote the $p_{r}$ regulators. Suppose that we have two measurements for the regulators, $p_{r_{1}}$ DM and $p_{r_{2}}$ CNA, then we can obtain $R_{n\times p_{r}}$ by stacking the measurements together with $p_{r}=p_{r_{1}}+p_{r_{2}}$. Next, we describe the overall analysis framework and integrative model.

### 2.1. Analysis Framework

First, consider a G×E model in the multi-omics scenario, where the regulators of the *G* factors are also included, in addition to the main and interaction effects.

(1) $$\begin{array}{cc} Y & =\sum_{k=1}^{q}\alpha_{k}E_{k}+\sum_{j=1}^{p_{g}}\left(\beta_{j}G_{j}+\sum_{k=1}^{q}\eta_{jk}G_{j}E_{k}\right)+\sum_{t=1}^{p_{r}}\gamma_{t}R_{t}+\epsilon , \end{array}$$

where $\alpha_{k}$, $\beta_{j}$, and $\eta_{jk}$ are the regression coefficients for the *k*th environmental factor, *j*th gene expression and their interactions, respectively. Besides, $\gamma_{t}$ is the regression coefficient for the *t*th regulator and ϵ is the random error.

Model (1) shares the spirit of parallel integration by treating the genetic factor and its regulators equally. Although such a strategy has shown to be effective in several studies, a more attractive alternative is to conduct vertical integration via accounting for the regulatory information among the different levels of omics measurements [5]. Typically, integrating multi-omics data in a main effect model with prognostic outcomes consists of two steps. At the first step, the sparse regulatory relationship can be identified, which leads to gene expressions that are modulated and not modulated by regulators, which can then be linked to clinical outcomes at the second step [6,8]. Specifically, Zhu et al. [6] proposed the linear regulatory model (LRM) to pinpoint the set of regulators that affect the corresponding set of GEs. Subsequently, the clinical model incorporates the GEs, residual GEs, and residual regulators. In this study, we extend the LRM to investigate the G×E interactions in the presence of multi-level omics measurements. In particular, the prognostic model at the second stage consists of: (1) a low dimensional environmental factors; (2) regulated GEs in the form of LRMs from the first stage and their interactions with those environmental factors; (3) Residual GEs and their interactions with environmental factors; and, (4) the residual effects of regulators.

### 2.2. Stage 1: The Linear Regulatory Model (LRM)

Denote $g=(g_{1},\cdots ,g_{p_{g}})$ as the $p_{g}$ gene expressions and denote $r=(r_{1},\cdots ,r_{p_{r}})$ as the $p_{r}$ regulators. The LRM can be expressed as

(2) $$\begin{array}{c} E\left(gV_{p_{g}\times L}|r\right)=a_{1\times L}+rU_{p_{r}\times L}, \end{array}$$

where *a* is the intercept, $V=(v_{1},\cdots ,v_{L})$ and $U=(u_{1},\cdots ,u_{L})$ both contain *L* columns of loading vectors ($v_{l}$ and $u_{l}$ for l∈{1,⋯,L}). Denote *L* as the total number of LRMs. Here, we assume *U* and *V* have orthogonal columns, such that $u_{l}⊥u_{l^{⊤}}$, $v_{l}⊥v_{l^{⊤}}$, for $l\neq l^{⊤}$. With this assumption, no overlap between gene expressions and regulators exists in LRM. We expect that different LRMs represent different regulated relationship between gene expressions and regulators [9]. In addition, $v_{l}$ and $u_{l}$ are assumed as sparse loading vectors, as only a small number of gene expressions is regulated by, at most, a small number of regulators [10].

For the *j*th gene expression, $j=1,\cdots ,p_{g}$, we right multiply $V^{⊤}$ to both sides in order to simplify Equation (2). Afterwards, the LRM can be formulated as a regression model with response variable $g_{j}$ and predictors *r*:

(3) $$\begin{array}{c} E\left(g_{j}\right)=a_{j}^{⊤}+r\theta_{j},\;\mathrm{for}\;j=1,\cdots ,p_{g}, \end{array}$$

where $a_{j}^{⊤}$ is an intercept and $\theta_{j}$ is the regression coefficient vector. Equation (3) indicates that one gene expression is regulated by a number of regulators. We impose sparsity on $\theta_{j}$ through penalization to identify a sparse regulatory relationship. Subsequently, the penalized regression model can be written as

(4) $$\begin{array}{c} \frac{1}{2n}∥g_{j}-a_{j}^{⊤}-r\theta_{j}{∥}_{2}^{2}+\lambda \left|\theta_{j}\right|,\;\mathrm{for}\;j=1,\cdots ,p_{g}, \end{array}$$

where λ is the tuning parameter. The LASSO is adopted for its computational simplicity and satisfactory performance [11]. Equation (4) leads to a regularized estimate of $\theta_{j}$, which indicates that one gene expression is regulated by a limited amount of regulators.

Next, we further investigate the relationship between sets of gene expressions and regulators through singular value decomposition (SVD). The regression model (3) can be collectively written as

(5) $$\begin{array}{c} E\left(g\right)=a^{⊤}+r\Theta_{p_{r}\times p_{g}} \end{array}$$

where $a^{⊤}$ is the vector of the intercept, $g_{1\times p_{g}}=\left(g_{1},\cdots ,g_{p_{g}}\right)$, $r_{1\times p_{r}}=\left(r_{1},\cdots ,r_{p_{r}}\right)$, and $\Theta_{p_{r}\times p_{g}}=\left(\theta_{1},\cdots ,\theta_{p_{g}}\right)$ is the transition matrix. The SVD is performed on the transition matrix in order to separate the regression coefficients representing gene expression and regulators:

(6) $$\begin{array}{c} \Theta =UDV^{⊤}=\left(u_{1},\cdots ,u_{L}\right)D{\left(v_{1},\cdots ,v_{L}\right)}^{⊤} \end{array}$$

where $D=\mathrm{diag}(d_{1},\cdots ,d_{L})$ is a diagonal matrix with *L* diagonal elements. The diagonal matrix *D* can account for the dissimilarity among loading vectors in terms of different scaling factors. Subsequently, we can obtain the estimated coefficients for gene expression and regulators by decomposing the estimated transition matrix $\hat{\Theta }$. Under the sparse condition, one gene expression is only regulated by a few of regulators, and one regulator affects a few of gene expressions [10]. In order to impose sparsity, we adopt the sparse SVD method that was developed by Lee et al. (2010) [12], where sparse singular vectors that correspond to the largest singular values are recursively obtained. Consider the first largest singular value $\left(d_{1},u_{1},v_{1}\right)$, then the regularized sparse SVD can be expressed as

(7) $$\begin{array}{c} \frac{1}{2n}∥\hat{\Theta }-d_{1}u_{1}v_{1}{∥}_{F}^{2}+\lambda |d_{1}u_{1}\left|+\lambda \right|d_{1}v_{1}| \end{array}$$

where ${∥\cdot ∥}_{F}$ is the Frobenius norm. Tuning parameter λ is the same for $u_{1}$ and $v_{1}$ for computation efficiency. Here $d_{1}$ is treated as the scaling factor. After estimating $\left(d_{1},u_{1},v_{1}\right)$, we update $\hat{\Theta }=\hat{\Theta }-{\hat{d}}_{1}{\hat{u}}_{1}{\hat{v}}_{1}^{⊤}$ and recursively update $\left(d_{l},u_{l},v_{l}\right)$, for l=2,⋯,L in a similar manner. With sparse SVD, we can decompose the coefficient and impose sparsity on $p_{z}$ and $p_{x}$ for every LRM. The standard LASSO is not applicable within the current LRM formulation, since the shrinkage has been imposed on scaled singular vectors.

### 2.3. Stage 2: The Penalized G×E Interaction Model

Now, we integrate multiomics measurements for G×E interactions. The regulated GEs, residual GEs, as well as residual regulators can be obtained through LRMs. The *G* factors are represented by regulated GEs and residual GEs, which are involved in the interaction with dimensional environmental factors. The partition of gene expressions into regulated and non-regulated components proceeds, as follows. The *L* sets of regulated gene expressions (GV) are equivalent to the corresponding sets of regulators (RU). We include the *L* sets of regulated GEs (GV) in the G×E model, since gene expressions are more directly related to cancer outcomes. The residual GEs, i.e., the non-regulated GEs that cannot be captured by LRMs, is denoted as ${\tilde{G}}_{n\times p_{g}}$. The G factors, consisting of both GV and $\tilde{G}$, interact with *q* environmental factors. Denote $W_{j}=\left(G_{j}V_{j},G_{j}V_{j}E_{1},\cdots ,G_{j}V_{j}E_{q},{\tilde{G}}_{j},{\tilde{G}}_{j}E_{1},\cdots ,{\tilde{G}}_{j}E_{q}\right)$, ($j=1,\cdots ,p_{g}$). Subsequently, $W_{j}$ corresponds to the interaction with respect to the *j*th GE. We only consider the main effect of residual regulators, because the influences of regulators on cancer outcomes are mostly mediated by gene expressions, and investigating its interactions with environmental factors is not of interest.

The quantifications of the residuals $\tilde{G}$ and $\tilde{R}$ are conducted through perpendicular projection operation. Because both can be calculated in the same manner, we take $\tilde{G}$ as an example. For the *j*th gene expression, define $S_{j}$ as the set of all LRMs that contains the *j* th gene expression. If $S_{j}$ is empty, then the *j*th gene expression is not regulated, which results in ${\tilde{G}}_{j}=G_{j}$. If $S_{j}$ is not empty, we denote $V_{S_{j}}$ as the sub-matrix of *V* that only contains columns (LRMs) of the *j*th gene expression. Following the perpendicular projection operation, we calculate the residual as ${\tilde{G}}_{j}=\left(I-GV_{S_{j}}{\left({\left(GV_{S_{j}}\right)}^{⊤}\left(GV_{S_{j}}\right)\right)}^{-1}{\left(GV_{S_{j}}\right)}^{⊤}\right)G_{j}$, which is the projection of $G_{j}$ onto the orthogonal space of $GV_{S_{j}}$.

Consider *n* subjects, $p_{g}$ gene expressions, and *L* LRMs. Subsequently, all of the main and interaction effects can be collectively written as

$$W=\left(GV,GVE_{1},\cdots ,GVE_{q},\tilde{G},\tilde{G}E_{1},\cdots ,\tilde{G}E_{q}\right)=\left(X_{1},X_{2}\right),$$

where $X_{1}=\left(GV,GVE_{1},\cdots ,GVE_{q}\right)$ denotes the main effects of regulated GEs and their interactions with the environmental factors. Similarly, the effects that correspond to residual GEs are defined as $X_{2}=\left(\tilde{G},\tilde{G}E_{1},\cdots ,\tilde{G}E_{q}\right)$. Subsequently, we consider the following penalized regression models for G×E interactions:

(8) $$\begin{array}{cc} \; & \frac{1}{2n}{∥Y-\sum_{k=1}^{q}\alpha_{k}E_{k}-\sum_{l=1}^{L}X_{1l}b_{1l}-\sum_{j=1}^{p_{g}}X_{2j}b_{2j}-\sum_{t=1}^{p_{r}}\gamma_{t}{\tilde{R}}_{t}∥}_{2}^{2}\\ \; & \;\;\;\;\;\;\;+\sum_{l=1}^{L}P_{1}\left(b_{1l};\lambda_{1}\right)+\sum_{j=1}^{p_{g}}P_{2}\left(b_{2j};\lambda_{2}\right)+\sum_{t=1}^{p_{r}}P_{3}\left(\gamma_{t};\lambda_{3}\right) \end{array}$$

where $X_{1l}=\left(GV_{l},GV_{l}E_{1},\cdots ,GV_{l}E_{q}\right),\;\left(l=1,\cdots ,L\right)$ represents the *l*th LRM and its interaction with *q* environmental factors, and $X_{2j}=\left({\tilde{G}}_{j},{\tilde{G}}_{j}E_{1},\cdots ,{\tilde{G}}_{j}E_{q}\right),\;\left(j=1,\cdots ,p_{g}\right)$ denotes the main and interaction effects with respect to the *j*th residual GEs. Here, $b_{1l}$ and $b_{2j}$ are the corresponding regression coefficients for $X_{1l}$ and $X_{2j}$. $\gamma_{t}$ is the coefficients for ${\tilde{R}}_{t}$ ($t=1,\cdots ,p_{r}$), the residual of regulators. $P_{i}\left(\cdot ;\lambda_{i}\right)$, (i=1,2,3), is the penalty function with $\lambda_{i}$ as the tuning parameter to impose sparsity. The three tuning parameters are set as the same because regression coefficients from the three components are on a similar scale, and different tunings dramatically increase the computational cost. Regularized identification in G×E interaction studies demands tailored penalty functions [7]. For instance, $b_{1l}$ stands for all the main and interaction effects with respect to the *l*th LRM. The selection of $b_{1l}$ on the group levels determines whether the *l*th LRM has any effect at all. If so, then selection of the individual effects within the group further determines the main and/or interactions that are associated with the cancer outcome. Therefore, penalized selection should accommodate the bi-level (or sparse group) structure. To be consistent with the analysis in stage 1, we still adopt LASSO as the baseline penalty function. Specifically, we have

$$\begin{array}{c} P_{1}\left(b_{1l};\lambda_{1}\right)=\lambda_{1}∥b_{1l}{∥}_{2}+\lambda_{1}\sum_{k=1}^{q+1}|b_{1lk}|,\;P_{2}\left(b_{2j};\lambda_{2}\right)=\lambda_{2}∥b_{2j}{∥}_{2}+\lambda_{2}\sum_{k=1}^{q+1}|b_{2jk}|, \end{array}$$

where $P_{1}\left(b_{1l};\lambda_{1}\right)$ and $P_{2}\left(b_{2j};\lambda_{2}\right)$ are sparse group LASSO. The L1 norm and L2 norm (${∥\cdot ∥}_{2}$) result in penalized identification on the individual and group level, respectively. The sparse group regularization has been adopted for the bi-level selection of main and interaction effects on the individual and group level simultaneously. Its advantage over LASSO in G×E studies has been demonstrated in multiple studies [7]. A corresponding price paid is computational cost, as different bi-level regularization usually demands different tunings. Because we only consider the main effect of residuals of regulators, the L1 norm penalty is adopted for $\gamma_{t}$ ($t=1,\cdots ,p_{r}$). Because the number of environmental factors is usually low, the selection of them is not of interest. They are pre-determined with evidence of being associated with cancer from previous studies. The proposed regularization respects a weak hierarchy between main and interaction effects as the penalty has not been imposed on the environmental main effects. Accordingly, once an interaction effect is selected, at least one of the two corresponding main effects will be in the model.

### 2.4. Computation

The Equation (8) can be expressed as:

(9) $$\begin{array}{c} \frac{1}{2n}∥Y-E\alpha -X_{1}b_{1}-X_{2}b_{2}-\tilde{R}\gamma {∥}_{2}^{2}+P_{1}\left(b_{1};\lambda_{1}\right)+P_{2}\left(b_{2};\lambda_{2}\right)+P_{3}\left(\gamma ;\lambda_{3}\right) \end{array}$$

where $\alpha_{q\times 1}={\left(\alpha_{1},\cdots ,\alpha_{q}\right)}^{⊤}$ is the coefficient vector for *q* environmental factors, $b_{1_{L(q+1)\times 1}}={\left(b_{1_{1}},\cdots ,b_{1_{L}}\right)}^{⊤}$ and $b_{2_{p_{g}\left(q+1\right)\times 1}}={\left(b_{2_{1}},\cdots ,b_{2_{p_{g}}}\right)}^{⊤}$ are the coefficient vectors for the main and interaction effects of the regulated and residual GEs, respectively. In addition, $\gamma_{p_{r}\times 1}=\left(\gamma_{1},\cdots ,\gamma_{p_{r}}\right)⊤$ is the coefficient vector for residual regulators.

The integrative analysis consists of two steps. In the first step, the loading matrices U and V are estimated through the construction of LRMs. The *j*th column of $\hat{\Theta }$, which is denoted as ${\hat{\theta }}_{j}$, ($j=1,\cdots ,p_{g}$), is estimated by minimizing Equation (4). For l=1,⋯,L, the singular vectors that correspond to the largest singular values, (${\hat{u}}_{l},{\hat{v}}_{l},{\hat{d}}_{l}$), are conducted through the rank-1 sparse SVD on $\hat{\Theta }$. The rank-1 sparse SVD is recursively performed for l=1,⋯,L, by updating ${\hat{\Theta }}^{\left(l+1\right)}={\hat{\Theta }}^{\left(l\right)}-{\hat{u}}_{l}{\hat{d}}_{l}{\hat{v}}_{l}^{⊤}$ at each *l*. In the second step, the shrinkage estimate of the regression coefficients can be obtained in the G×E model, where GV, RU, residuals of gene expressions ($\tilde{G}$), and residuals of regulators ($\tilde{R}$) are calculated accordingly. At the *k*th iteration, the vector of estimated regression coefficients for all of the environmental factors is computed by ${\hat{\alpha }}^{\left(k+1\right)}={\left(E^{{\left(k\right)}^{⊤}}E^{\left(k\right)}\right)}^{-1}E^{{\left(k\right)}^{⊤}}\left(Y-X_{1}{\hat{b}}_{1}^{\left(k\right)}-X_{2}{\hat{b}}_{2}^{\left(k\right)}-\tilde{R}{\hat{\gamma }}^{\left(k\right)}\right)$. Given ${\hat{\alpha }}^{\left(k+1\right)}$ fixed at the current estimate, we obtain $\left({\hat{b}}_{1}^{\left(k+1\right)},{\hat{b}}_{2}^{\left(k+1\right)},{\hat{\gamma }}^{\left(k+1\right)}\right)$ by minimizing Equation (9). The iteration stops until convergence. Algorithm 1 shows the outline of algorithm:
**Algorithm 1** The Integrative analysis for G×E Interaction**Step 1:** Estimate the loading matrices of LRMs U and V: construct LRMs.(a) For $j=1,\cdots ,p_{g}$, obtain ${\hat{\theta }}_{j}$ by minimizing Equation (4). Then the estimate $\hat{\Theta }=\left({\hat{\theta }}_{1},\cdots ,{\hat{\theta }}_{p_{g}}\right)$.Initialize l=1.**for**l=1,⋯,L**do** (b) Apply rank-1 sparse SVD on $\hat{\Theta }$ to obtain the singular vectors corresponding to largest singular values $\left(u_{l},v_{l},d_{l}\right)$. (c) Update ${\hat{\Theta }}^{\left(l+1\right)}={\hat{\Theta }}^{\left(l\right)}-u_{l}d_{l}v_{l}^{⊤}$. (d) l=l+1.**end for****Step 2:** Estimate regression coefficients $\alpha ,b_{1},b_{2},\gamma$: construct the penalized G×E interaction model.(a) Calculate GV, RU, $\tilde{G}$ and $\tilde{R}$.Initialize ${\hat{b}}_{1}^{\left(0\right)}={\hat{b}}_{2}^{\left(0\right)}={\hat{\gamma }}^{\left(0\right)}=0$.At the (k+1)th iteration.**repeat** (b) Compute ${\hat{\alpha }}^{\left(k+1\right)}={\left(E^{{\left(k\right)}^{⊤}}E^{\left(k\right)}\right)}^{-1}E^{{\left(k\right)}^{⊤}}\left(Y-X_{1}{\hat{b}}_{1}^{\left(k\right)}-X_{2}{\hat{b}}_{2}^{\left(k\right)}-\tilde{R}{\hat{\gamma }}^{\left(k\right)}\right)$. (c) Obtain $\left({\hat{b}}_{1}^{\left(k+1\right)},{\hat{b}}_{2}^{\left(k+1\right)},{\hat{\gamma }}^{\left(k+1\right)}\right)$ by minimizing Equation (9) through bi-level selection.**until** convergence

LASSO is adopted in order to conduct the selection of important LRMs from the first stage. At the second stage, a sparse group LASSO has been formulated to accommodate the identification of main and interaction effects on both the group and individual level. We conjecture that other penalization methods, such as adaptive LASSO [13], SCAD [14], and MCP [15], are also applicable in our framework. For example, MCP can be adopted in order to identify sparse regulatory relationship from the first stage, and a sparse group MCP is also tailored for the identification of important G×E interactions in the clinical model. We do not compare the performances of different baseline penalization methods within our framework, as it is not the main interest here.

At the first step, we only use one tuning parameter λ for conducting sparse SVD, due to the similarity in scales between GE and its regulators. The three tuning parameters, $\lambda_{1},\lambda_{2},\lambda_{3}$, have been used in the second step, where $\lambda_{1}$ and $\lambda_{2}$ determine the sparsity of main and interaction effects with respect to the regulated and unregulated GEs correspondingly, and $\lambda_{3}$ controls the sparsity of the residuals from regulators. We choose the optimal tuning parameters using five-fold cross-validation in both the simulation study and real data analysis. The analysis has been implemented with statistical software R (version 3.6.3). In simulation, the average CPU time of running one replicated simulated data $\left(n=500,\;p_{g}=p_{r}=200,\;q=4\right)$ is 23.1 min. on a regular desktop PC. The R codes are available from the corresponding author.

## 3. Simulation

We perform simulation in order to evaluate the utility of the proposed method integrative G×E model, termed IGE. In addition, we consider three alternative methods: (1) the S-LASSO selects gene expressions and regulators separately using LASSO. (2) The J-LASSO selects gene expressions and regulators that are based on LASSO simultaneously. (3) ColReg, the collaborative regression [16], identifies important GEs and regulators jointly in terms of explaining similar variation under the cancer outcome.

We generate the data, as follows. First, each row of *R* is independently generated from a multivariate normal distribution with mean zero and one of the four covariance structures: (i) AR–1 structure with correlation coefficient $0.25^{\left|i-j\right|}$ for the *i*th and *j*th regulators; (ii) banded correlation structure, where the *i*th and *j*th regulators have ρ=0.33 if |i−j|=1 and ρ=0 otherwise; (iii) the covariance that was extracted from TCGA lung squamous cell carcinoma (LUSC) data in Section 4; and, (iv) the covariance structure of the lung adenocarcinoma (LUAD) from Section 4.

Choose L=20 for the number of LRMs between gene expression and regulators. For l=1,⋯,20, $u_{l}$ or $v_{l}$ is randomly assigned five non-zero entries, with values being generated from unif [2,4]. Subsequently, Θ is computed as $\sum_{l=1}^{20}u_{l}v_{l}^{⊤}$ and *G* is generated as G=RΘ+ε, where each row of matrix ε is independently generated from a multivariate normal distribution with mean zero and the same covariance structure as *R*. To generate the cancer outcome, each row of *E* is generated independently from a multivariate normal distribution with marginal mean zero and AR-1 structure, where the *i*th and *j*th components have correlation coefficient $0.5^{\left|i-j\right|}$. Subsequently, we generate the response from model (1) under standard normal errors.

200 gene expression, 200 regulators, and four environmental factors are simulated with two different sample sizes, 500 and 1000. We randomly select 30 gene expressions to assign non-zeros effects in model (1). For every selected gene expression, four non-zero entries are randomly assigned to the coefficients of G factor or its corresponding G×E interactions. Those values are generated from unif [0.25,0.5] and unif [0.5,1] for weak and strong coefficient signals, respectively. The coefficients of regulators are randomly assigned with 30 non-zero coefficients being generated from unif [1,2]. The coefficients of environmental factors are generated from unif [2,3].

**Figure 1:**
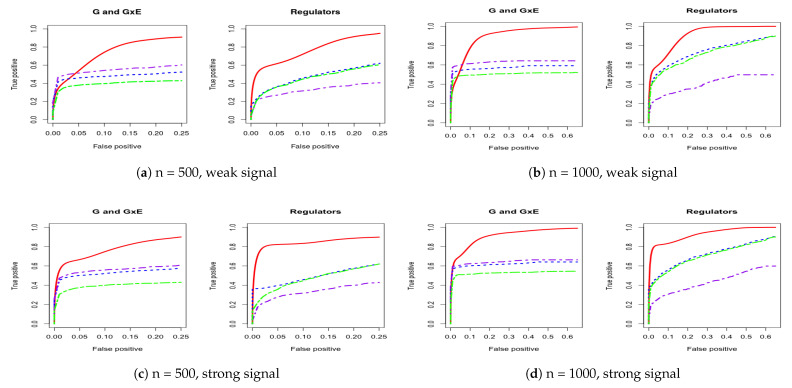
Four cases of receiver operating characteristic (ROC) curves under AR-1 structure. The left panel corresponds to comparison under both weak and strong signals for 500 subjects. The right panel corresponds to comparison under both weak and strong signals for 1000 subjects. IGE, solid red; S-LASSO, dashed blue; J-LASSO, long dashed purple; ColReg, long dashed green.

**Figure 2:**
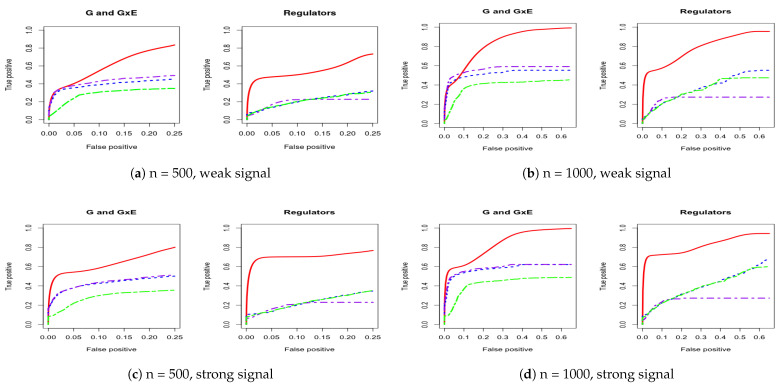
Four cases of ROC curves under estimated covariance from lung squamous cell carcinoma (LUSC). The left panel corresponds to comparison under both weak and strong signals for 500 subjects. The right panel corresponds to comparison under both weak and strong signals for 1000 subjects. IGE, solid red; S-LASSO, dashed blue; J-LASSO, long dashed purple; ColReg, long dashed green.

For a comprehensive evaluation, we consider a sequence of tuning parameter values (from 0 to 3, total 100 lambda values) and then use the receiver operating characteristic (ROC) curve and partial area under the ROC curve (PAUC) to compare the different methods. The total simulation replication is 100. All of the PAUCs are tabulated in Table 1 and Table 2. Figure 1 and Figure 2 show the ROC curves for the AR-1 structure and estimated covariance from LUSC. Appendix A provides other scenarios of ROC curves, respectively.

We consider using the receiver operating characteristic (ROC) curve and the partial area under the ROC curve (PAUC) to compare different methods. Total simulation replicates is 100. Table 1 and Table 2 tabulate all of the PAUCs. Figure 1 and Figure 2 show the ROC curves for AR-1 structure and estimated covariance from LUSC. Appendix A provides the ROC curves in other scenarios. For all simulation scenarios, the proposed method has higher PAUCs than the alternative methods. For example, in Table 1 with AR-1 correlation and weak signal, the proposed method has PAUC 0.73 (sd 0.07) for the identification of G and G×E effects, while J-LASSO, S-LASSO, and ColReg have PAUCs 0.54 (sd 0.04), 0.47 (sd 0.04), and 0.39 (sd 0.03), respectively. For the identification of regulators, the proposed method has PAUC 0.76 (sd 0.10), while J-LASSO, S-LASSO, and ColReg have PAUCs 0.32 (sd 0.05), 0.46 (sd 0.13), and 0.45 (sd 0.15), respectively. The similar pattern can be observed under settings with strong signals. When the sample size increases, the identification results of all methods become better. The proposed IGE outperforms alternative approaches across different scenarios. For instance, in Table 2 with AR-1 correlation and strong signal, the proposed method has PAUC 0.89 (sd 0.02) in the identification of G and G×E, while J-LASSO, S-LASSO, and ColReg have PAUCs 0.62 (sd 0.04), 0.57 (sd 0.04), and 0.50 (sd 0.03), correspondingly. For the identification of regulators, the proposed method also outperforms the alternatives.

In addition, the proposed method outperforms the alternatives when the correlation is extracted from real data. For example, in Table 1, with estimated covariance from LUSC and weak signals, the proposed method has close PAUCs in both G and G×E and regulators, 0.59 (sd 0.09) and 0.55 (sd 0.15). Other methods have low accuracy in identifying main and interaction effects. In particular, J-LASSO, S-LASSO, and ColReg have PAUCs 0.42 (sd 0.05) and 0.19 (sd 0.06), 0.39 (sd 0.04) and 0.21 (sd 0.06), and 0.28 (sd 0.04) and 0.21 (sd 0.07), respectively. When magnitude of the signals and sample size increase (e.g., with LUSC and strong signals), the proposed method still have the best performance in identification. Overall, the IGE model has much higher identification accuracy than other methods across different simulation settings by borrowing strength from accounting for regulatory relationship and bi-level selection in G×E interaction studies.

## 4. Analysis of TCGA Data

Lung cancer is a top rank common cancer for both men and women. In this section, we apply the proposed method as well as the alternatives on lung adenocarcinoma (LUAD) data and lung squamous cell carcinoma (LUSC) data from the Cancer Genome Atlas (TCGA, https://cancergenome.nih.gov/).

At present, LUAD is the most common lung cancer subtype among non-smokers and women, although it has been shown that smoking may increase the risk of LUAD [17,18]. On the other hand, LUSC is closely associated with smoking, and it is more common in men than in women [19]. LUAD grows more slowly with smaller masses than LUSC of the same stage, but LUAD tends to initiate metastasis at the early stages [20].

The processed level 3 data have been downloaded from TCGA data portal while using package *cgdsr*. We match the multi-omics measurements with the clinical/environmental variables and survival outcome. LUSC and LUAD has 344 and 426 subjects, correspondingly. We first conduct screenings to reduce dimensionality, so the regularization methods can be appropriately applied. Here, we select the top 200 mRNA with the largest marginal variances. As we matched the CNA and Methylation profiles with same mRNA, the corresponding 200 measurements on CNA and Methylation are selected at the same time. We select age, gender, smoking pack years, and pathologic tumor stage as environmental variables. The accelerated failure time (AFT) model (Appendix B) has been adopted in order to link the omics and clinical measurements to survival outcomes.

### 4.1. Lung Adenocarcinoma (LUAD) Data

The proposed method identifies eight LRMs with one residual effect of gene expression (mRNA) and 14 residual effects of regulators (DM and CNA). Additionally, the proposed method results in the identification of seven LRM×E interactions and 11 G×E interactions from mRNA residual effects.

Table 3 provides the identified main effects of LRMs, residual GEs, and regulators. We can observe that LRMs does not contain effects from methylation, while most of the residual effects in regulators are from methylation.The identification results have important biological implications. As a representative example, gene PIK3R2 is identified by 6 different LRMs. From a recent study [21], PIK3R2 is significantly associated with lung adenocarcinoma and its pathway plays a critical role in the progress of LUAD. Besides, gene STK3 is identified by five different LRMs. STK3 belongs to a large family of serine/threonine kinases, which are implicated in the regulation of signaling pathways involved in cell growth, differentiation and death. [22,23]. The identified LRMs are also meaningful. For example, we observe the regulatory relationship between PIK3R2 and NEK2 from both LRM #1 and #6. One of the recent studies shows that this natural downstream regulation is significantly related to cancer outcome [24]. Among all of the residual effects, we observe that most of them are from methylation. For example, SLC2A1, ECT2, TNS4, DKK1, and GNPNAT1 are found to be associated with the survival of lung cancer patients [25,26,27,28,29].

Table 4 provides the identification results for interaction effects. The proposed method selects variables with a sparse group nature. There are five LRMs interacting with environments. The first and fourth LRMs interact with two environment factors, and the second, third, and fifth interact with one environment factor. Additionally, the proposed method can identify a total of 11 interactions involving mRNA residual effects. Note that, here, the G factor is no longer in the usual sense from existing G×E studies. The G factor are represented by the LRMs and residual mRNAs that correspond to the regulated and un-regulated G factors, respectively.

In terms of prediction, we adopt a random sampling approach. More specifically, we randomly select 30% data as a test set and the remaining as a training set. The estimates are generated using the training set only and the predictions are made based on the testing set. We dichotomize the predicted response at the median, create two risk groups, and compute log-rank statistics, which measure the difference in survival between the two groups. Larger log-rank test statistic indicates better predictive performance. The procedure is repeated 100 times to avoid extreme splits. The average log-rank test statistics are 5.97 (IGE, sd 0.35), 4.76 (S-LASSO, sd 0.25), 4.60 (J-LASSO, sd 0.08), and 3.74 (ColReg, sd 0.26), respectively. The proposed method has the largest log-rank statistic, hence the best prediction performance.

### 4.2. Lung Squamous Cell Carcinoma (LUSC) Data

The proposed method identifies eight LRMs with two residual effects from GEs and 17 residual effects from regulators (DM and CNA). The interactions involve seven LRMs and 26 mRNAs.

Table 5 provides the identified main effects using the proposed method. As aforementioned, we aim to find a sparse relationship between gene expressions and regulators. Therefore, a small subset of regulators are related to genes and vice versa. Table 6 provides the identifications of G×E interaction effects. There’s one LRM not interacting with any other environmental factors. The findings have important implications. For instance, gene RNF24 is identified by 2 different LRMs (#1, #2). RNF24 is a membrane protein, which interacts with TRPC protein [30]. A recent study shows that RNF24 acts as one of the important factors for the prognosis of carcinoma [31]. RNF24 is also shown to be correlated with the occurrence of esophageal adenocarcinoma [32]. For DM, RGP1 is identified by three different LRMs (#4, #6, #7). RGP1 belongs to the regulation of guanosine diphosphate (GDP) reaction exchange, and it acts as a prognostic factor in cancer, according to Anand (2020) [33]. For CNA, CD163L1 is identified by three different LRMs (#1, #4, #8), and it can be used as a significant biomarker of cancer [34]. The identified LRMs are also meaningful. For example, the regulatory relationship between NCOR2 and TCTN2 can be identified in LRM #7. This result has also been observed in a regulatory network analysis [35]. Among all of the residual effects, LRAT, PLEKHA6, ACOT7, KLK6, PLEKHB1, FGFRL1, and FPR2 are associated with prognosis of LUSC patients from existing studies [36,37,38,39,40,41].

We adopt a random sampling approach and apply log-rank test for assessment in order to evaluate prediction. We adopt the similar procedure as previous real data analysis section. After repeating 100 times, the average log-rank test statistics are 33.20 (IGE, sd 2.32), 25.06 (S-LASSO, sd 1.84), 24.41 (J-LASSO, sd 2.13), and 27.88 (ColReg, sd 2.45), respectively. The proposed method has superior prediction performance over alternatives.

## 5. Discussion

We have conducted an integrative gene–environment interaction analysis for multi-dimensional omics data based on the proposed two-step variable selection model. Specifically, at the first step, sparse regulatory relationship between the G factor and its regulators have been pinpointed via penalization, which leads to effects that can be directly linked to the prognostic outcomes. At the second step, a G×E prognostic model has been considered, where the G factor that is involved in the interaction consists of regulated (corresponding to the LRM) and unregulated (i.e., the residual GE) components. Besides, the residuals of the regulator are also included. The integrative G×E analysis fully takes the advantage of the multi-omics measurements, which distinguishes itself from most of the published studies.

Traditionally, statistical testing based marginal analysis has dominated the G×E studies. The paradigm shift to the joint analysis has been mainly motivated by the gene set and pathway-based association analysis [42,43,44,45]. Recently, the effectiveness of regularized variable selection has been recognized not only in joint G×E studies when a large number of genetic factors are involved [7], but also in multi-level omics integrations [5]. Therefore, it has been adopted here.

This study can be improved by the following aspects. Because strong correlations have been widely observed in among omics measurements, network based penalization can be imposed to accommodate the correlations among regulators at the first stage [46,47,48]. Besides, robustness can be incorporated at the first stage to model the regulatory relationship between GE and its regulators [49], and in the second stage for a robust prognostic model [50,51]. Accounting for the form of environmental factors has received considerable attention in G×E studies, which results in the development of a wide range of nonparametric [52,53,54] and semiparametric [55,56,57] methods. However, in integrative G×E studies, capturing the nonlinear form of interaction is challenging. In this study, we focus on prognostic outcomes. With other types of outcomes, such as the longitudinal phenotypes [58,59], the G×E model in the second stage can be modified accordingly.

## Figures and Tables

**Table 1:** PAUC: mean (sd) based on 100 replicates. $p_{g}=p_{r}=200,n=500$.

Covariance	Signal	Approaches	G and G×E	Regulators
AR-1	weak	IGE	0.73 (0.07)	0.76 (0.10)
		S-LASSO	0.47 (0.04)	0.46 (0.13)
		J-LASSO	0.54 (0.04)	0.32 (0.05)
		ColReg	0.39 (0.03)	0.45 (0.15)
	strong	IGE	0.77 (0.07)	0.85 (0.06)
		S-LASSO	0.52 (0.05)	0.48 (0.14)
		J-LASSO	0.55 (0.04)	0.33 (0.05)
		ColReg	0.39 (0.03)	0.46 (0.15)
Banded	weak	IGE	0.74 (0.06)	0.74 (0.10)
		S-LASSO	0.48 (0.03)	0.44 (0.11)
		J-LASSO	0.54 (0.05)	0.32 (0.04)
		ColReg	0.39 (0.03)	0.43 (0.12)
	strong	IGE	0.77 (0.08)	0.84 (0.06)
		S-LASSO	0.52 (0.04)	0.46 (0.11)
		J-LASSO	0.55 (0.05)	0.32 (0.04)
		ColReg	0.39 (0.03)	0.43 (0.12)
LUSC	weak	IGE	0.59 (0.09)	0.55 (0.15)
		S-LASSO	0.39 (0.04)	0.21 (0.06)
		J-LASSO	0.42 (0.05)	0.19 (0.06)
		ColReg	0.28 (0.04)	0.21 (0.07)
	strong	IGE	0.63 (0.10)	0.71 (0.13)
		S-LASSO	0.42 (0.05)	0.22 (0.07)
		J-LASSO	0.43 (0.05)	0.19 (0.06)
		ColReg	0.28(0.05)	0.22 (0.07)
LUAD	weak	IGE	0.64 (0.09)	0.62 (0.15)
		S-LASSO	0.45 (0.04)	0.21 (0.06)
		J-LASSO	0.47 (0.05)	0.19 (0.05)
		ColReg	0.32 (0.03)	0.22 (0.07)
	strong	IGE	0.70 (0.08)	0.77 (0.11)
		S-LASSO	0.47 (0.05)	0.23 (0.08)
		J-LASSO	0.48 (0.05)	0.18 (0.05)
		ColReg	0.31 (0.04)	0.23 (0.08)

**Table 2:** PAUC: mean (sd) based on 100 replicates. $p_{g}=p_{r}=200,n=1000$.

Covariance	Signal	Approaches	G and G×E	Regulators
AR-1	weak	IGE	0.89 (0.02)	0.91 (0.02)
		S-LASSO	0.57 (0.04)	0.73 (0.09)
		J-LASSO	0.62 (0.04)	0.40 (0.04)
		ColReg	0.50 (0.03)	0.71 (0.09)
	strong	IGE	0.91 (0.02)	0.93 (0.02)
		S-LASSO	0.61 (0.04)	0.71 (0.08)
		J-LASSO	0.64 (0.05)	0.43 (0.04)
		ColReg	0.52 (0.03)	0.70 (0.09)
Banded	weak	IGE	0.89 (0.03)	0.91 (0.03)
		S-LASSO	0.55 (0.04)	0.73 (0.07)
		J-LASSO	0.62 (0.04)	0.40 (0.05)
		ColReg	0.50 (0.03)	0.71 (0.08)
	strong	IGE	0.90 (0.04)	0.92 (0.02)
		S-LASSO	0.61 (0.04)	0.72 (0.08)
		J-LASSO	0.64 (0.04)	0.44 (0.06)
		ColReg	0.53 (0.04)	0.70 (0.08)
LUSC	weak	IGE	0.82 (0.04)	0.78 (0.06)
		S-LASSO	0.51 (0.05)	0.36 (0.07)
		J-LASSO	0.56 (0.05)	0.25 (0.07)
		ColReg	0.39 (0.04)	0.35 (0.08)
	strong	IGE	0.83 (0.04)	0.82 (0.06)
		S-LASSO	0.57 (0.05)	0.39 (0.07)
		J-LASSO	0.58 (0.05)	0.25 (0.08)
		ColReg	0.42 (0.04)	0.38 (0.07)
LUAD	weak	IGE	0.83 (0.04)	0.80 (0.06)
		S-LASSO	0.57 (0.04)	0.43 (0.06)
		J-LASSO	0.59 (0.04)	0.25 (0.06)
		ColReg	0.47 (0.03)	0.43 (0.06)
	strong	IGE	0.85 (0.03)	0.84 (0.04)
		S-LASSO	0.61 (0.04)	0.46 (0.07)
		J-LASSO	0.61 (0.04)	0.26 (0.06)
		ColReg	0.49 (0.03)	0.46 (0.07)

**Table 3:** Analysis of the the Cancer Genome Atlas (TCGA) lung adenocarcinoma (LUAD) data: linear regulatory models (LRMs) and residual effects for gene expression and regulators with the estimated coefficient or loadings in the parentheses.

LRMs				
	#1 (0.07)	#2 (−0.01)	#3 (−0.02)	#4 (−0.03)
mRNA	PIK3R2 (0.35)	PIK3R2 (0.98)	ECT2 (−0.98)	INTS7 (−0.77)
	STK3 (−0.74)	STK3 (0.11)	PSMD2 (−0.17)	PIK3R2 (−0.62)
	NCKAP5L (0.74)	NCKAP5L (−0.08)		
	CUL9 (0.14)			
CNA	NEK2(−0.22)	CECR1 (0.65)	KPNA4 (−0.44)	INTS7 (−0.70)
	LPGAT1 (0.22)	C1QTNF6 (−0.75)	B3GALNT1 (0.43)	DTL (0.70)
	INTS7 (0.65)		PSMD2 (−0.55)	
	DTL (−0.65)		LIPH (0.55)	
	CECR1 (−0.19)			
	#5 (−0.05)	#6 (0.08)	#7 (−0.06)	#8 (0.06)
mRNA	PIK3R2 (0.12)	INTS7 (0.73)	PIK3R2 (−0.10)	PSMD2 (0.31)
	STK3 (−0.78)	PIK3R2 (0.63)	STK3 (−0.24)	TMOD 3(0.61)
	NCKAP5L (0.57)	STK3 (0.18)	CUL9 (−0.96)	DIAPH3 (0.72)
	CUL9 (0.16)	NCKAP5L (−0.14)		
CNA	INTS7 (−0.16)	NEK2 (−0.69)	INTS7 (−0.34)	MAPRE3 (0.70)
	DTL (0.16)	LPGAT1 (0.71)	DTL (0.36)	IFT172 (−0.67)
	CECR1 (−0.78)		CECR1 (0.61)	PSMD2 (0.09)
	C1QTNF6 (−0.57)		C1QTNF6 (−0.61)	ITGB1 (0.09)
				ADAM10 (0.14)
Residual effects				
mRNA	MAST3 (0.01)			
DM	ADSS (0.01)	SLC2A1 (0.01)	PTCH2 (0.01)	ECT2 (0.09)
	TNS4 (0.02)	MUSTN1 (0.05)	DKK1 (0.02)	FSCN1 (0.05)
	GNPNAT1 (0.04)	HPS1 (−0.04)	MAPRE3 (−0.02)	
CNA	LAMC2 (−0.01)	CD5 (−0.03)	E2F7 (−0.01)	

**Table 4:** Analysis of the TCGA LUAD data: G×E interaction identifications from LRMs and gene expression with the estimated regression coefficients in the parentheses.

LRMs	AGE	GENDER	SMOKING
#1	0.08		−0.25
#2		0.02	
#3		0.01	
#4		0.01	0.01
#5			0.01
mRNA Residual	AGE	GENDER	SMOKING
MAST3			0.27
HPS1	0.01		
BBS5	−0.04		−0.03
TLE1	−0.01		
ADAM10		0.02	0.03
SLC16A3		0.07	
BTN2A2		−0.02	−0.06
FAM71E1			0.02

**Table 5:** Analysis of the TCGA LUSC data: LRMs and residual effects for gene expression and regulators with the estimated coefficient or loadings in the parentheses.

LRMs				
	#1 (−0.01)	#2 (0.01)	#3 (0.01)	#4 (−0.02)
mRNA	RNF24 (−0.17)	SEC23B (0.23)	REEP3 (−0.76)	AP2A2 (−0.59)
	ESM1 (−0.53)	RNF24 (−0.97)	FUT11 (−0.64)	PNPLA6 (−0.37)
	RASAL2 (−0.39)			RFX1 (−0.55)
	LAMC1 (−0.34)			XRN2 (0.45)
	DLGAP4 (−0.63)			
DM	DCBLD1 (0.09)	TCF7L2 (0.22)		RGP1 (−0.52)
	CHI3L1 (0.18)			NCOR2 (0.27)
CNA	CD163L1 (−0.16)	ENTPD6 (0.68)	RERE (−0.89)	CD163L1 (0.70)
	DLGAP4 (−0.96)	ABHD12 (−0.69)	DLGAP4 (−0.43)	PARD6G (−0.39)
	#5 (0.16)	#6 (0.05)	#7 (−0.05)	#8 (0.01)
mRNA	COL5A3 (0.45)	MGST3 (0.33)	TPM4 (0.68)	TCTN2 (−0.45)
	DCBLD1 (0.57)	OSBPL5 (0.31)	UBB (0.59)	ANGPT2 (−0.40)
	PDGFA (0.31)	SNX9 (0.56)	NCOR2 (−0.42)	UBE4B (−0.37)
	CHST15 (0.45)	MYO1C (0.46)		MBTPS1 (−0.47)
	LGALS1 (0.39)	CCDC68 (0.49)		FAM178B (−0.50)
DM	DCBLD1 (−0.86)	CHST15 (−0.97)	RGP1 (−0.55)	NCOR2 (0.16)
	FAM178B (−0.37)	RGP1 (0.13)		
	CHST15 (−0.17)	NCOR2 (−0.10)		
		LGALS1 (−0.15)		
CNA	DLGAP4 (0.27)		STK40 (−0.26)	CD163L1 (−0.35)
			TCTN2 (−0.78)	DLGAP4 (−0.92)
Residual effects				
mRNA	LRAT (−0.02)	PLEKHA6 (−0.02)		
DM	BAMBI (0.01)	PYGB (0.02)	FUT11 (−0.18)	ZNF394 (0.03)
	CCIN (−0.01)	DEAF1 (−0.10)	ACOT7 (0.04)	KLK6 (−0.12)
	LHX8 (−0.01)	PLEKHB1 (0.09)		
CNA	FGFRL1 (−0.05)	DCBLD1 (−0.04)	NEFL (−0.04)	CHST1 (0.02)
	ULK1 (−0.03)	FPR2 (0.02)	PYGB (−0.10)	

**Table 6:** Analysis of the TCGA LUSC data: G×E interaction identifications from LRMs and gene expression with the estimated regression coefficients in the parentheses.

LRMs	AGE	GENDER	SMOKING
#1		0.02	0.03
#2		0.03	
#4	−0.02		
#5	0.01	0.05	−0.02
#6	0.01	−0.01	
#7		−0.36	
#8		0.02	
mRNA Residual	AGE	GENDER	SMOKING
LRAT		−0.17	
PLEKHA6		−0.30	
AP2A2	0.02		
SLC12A7	−0.10	0.07	
TCTN2	−0.15	−0.09	
CLEC5A	0.01		
RNF24	−0.06	0.04	
PRRX2	0.04		−0.04
CCDC74A	0.14	−0.13	
FGF9	0.03		−0.06
IGF2R	0.05	−0.02	
CHMP4C	0.24	0.13	−0.01
SLC45A4	−0.11		
SULF2	−0.05	−0.03	
UBB		−0.11	
DVL1		−0.07	
NID1		0.08	0.20
KLK8		0.01	
DOCK6		0.26	−0.10
FHDC1		0.01	−0.16
OPLAH		−0.12	
VSTM1			−0.02
SLC28A1			−0.07
TCF7L2			0.12
DLGAP4			−0.04
CRNKL1			−0.25

**Figure A1:**
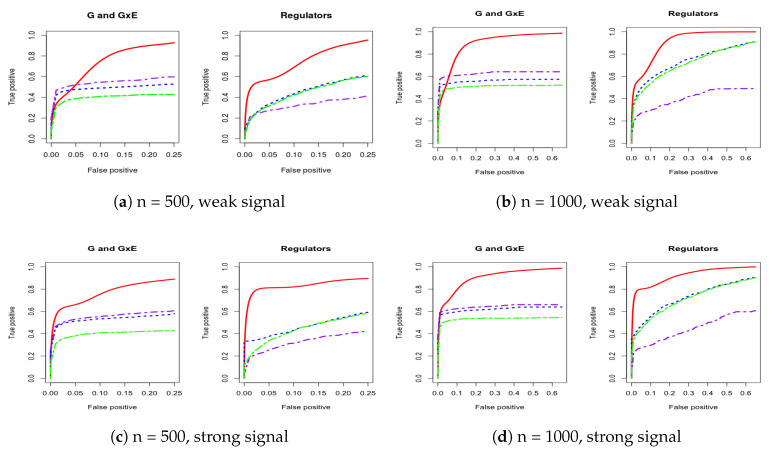
Four cases of ROC curves under banded correlation structure. Left two columns are 500 subjects to compare weak and strong signal performance. Right two columns are 1000 subjects to compare weak and strong signal performance. IGE, solid red; S-LASSO, dashed blue; J-LASSO, long dashed purple; ColReg, long dashed green.

**Figure A2:**
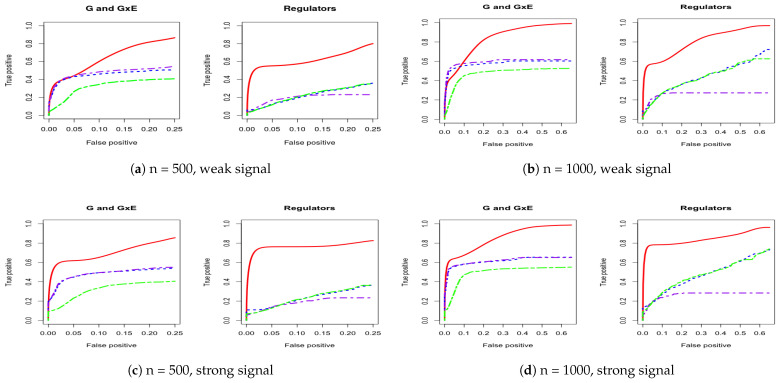
Four cases of ROC curves under estimated covariance from LUAD. Left two columns are 500 subjects to compare weak and strong signal performance. Right two columns are 1000 subjects to compare weak and strong signal performance. IGE, solid red; S-LASSO, dashed blue; J-LASSO, long dashed purple; ColReg, long dashed green.

## Data Availability

The datasets used for the analyses described in this manuscript has been downloaded from the TCGA data portal (https://portal.gdc.cancer.gov/) and are available to the general public without restricted access.

## Appendix B. Accelerated Failure Time (AFT) Model

Denote *T* as the logarithm of the failure time and denote *C* as the logarithm of the censoring time. Under right censoring, we observe Y=min(T,C),δ=I(T≤C). We adopt the Kaplan-Meier weights for censoring. Let $\hat{F}$ be the Kaplan-Meier estimator of the distribution function *F* of *T*. According to [60], we have $\hat{F}\left(y\right)=\sum_{i=1}^{n}w_{i}I\left\{Y_{\left(i\right)}\leq y\right\}$, where $w_{i}$ can be computed as

$$\begin{array}{c} w_{1}=\frac{\delta_{\left(1\right)}}{n},w_{i}=\frac{\delta_{\left(i\right)}}{n-i+1}\prod_{j=1}^{i-1}{\left(\frac{n-j}{n-j+1}\right)}^{\delta_{j}},\;i=2,...,n, \end{array}$$

where $Y_{\left(1\right)}\leq \cdots \leq Y_{\left(n\right)}$ are the order statistics of $Y_{i}$ and $\delta_{\left(1\right)},\cdots ,\delta_{\left(n\right)}$ are the corresponding censoring indicators. Denote $\left(E_{\left(i\right)},\;X_{1_{\left(i\right)}},\;X_{2_{\left(i\right)}},{\tilde{R}}_{\left(i\right)}\right)$ as the measurements associated with $\left(Y_{\left(i\right)},\delta_{\left(i\right)}\right)$, where the notations are from Equation (9). We center $E_{\left(i\right)}$, $X_{1_{\left(i\right)}}$, $X_{2_{\left(i\right)}}$, ${\tilde{R}}_{\left(i\right)}$, $Y_{\left(i\right)}$ using $w_{i}$-weighted mean as follows:

$$\begin{array}{c} {\bar{E}}_{w}=\sum_{i=1}^{n}w_{i}E_{\left(i\right)}/\sum_{i=1}^{n}w_{i},\;{\bar{X}}_{1_{w}}=\sum_{i=1}^{n}w_{i}X_{1_{\left(i\right)}}/\sum_{i=1}^{n}w_{i},{\bar{X}}_{2_{w}}=\sum_{i=1}^{n}w_{i}X_{2_{\left(i\right)}}/\sum_{i=1}^{n}w_{i}, \end{array}$$

$$\begin{array}{c} {\bar{\tilde{R}}}_{w}=\sum_{i=1}^{n}w_{i}{\tilde{R}}_{\left(i\right)}/\sum_{i=1}^{n}w_{i},\;{\bar{Y}}_{w}=\sum_{i=1}^{n}w_{i}Y_{\left(i\right)}/\sum_{i=1}^{n}w_{i}. \end{array}$$

Then the centered predictors and responses are $E_{w_{\left(i\right)}}=\sqrt{w_{i}}\left(E_{\left(i\right)}-{\bar{E}}_{w}\right),\;X_{1_{w_{\left(i\right)}}}=\sqrt{w_{i}}\left(X_{1_{\left(i\right)}}-{\bar{X}}_{1_{w}}\right),\;X_{2_{w_{\left(i\right)}}}=\sqrt{w_{i}}\left(X_{2_{\left(i\right)}}-{\bar{X}}_{2_{w}}\right),\;{\tilde{R}}_{w_{\left(i\right)}}=\sqrt{w_{i}}\left({\tilde{R}}_{\left(i\right)}-{\bar{\tilde{R}}}_{w}\right)\;\mathrm{and}\;Y_{w_{\left(i\right)}}=\sqrt{w_{i}}\left(Y_{\left(i\right)}-{\bar{Y}}_{w}\right).$ Hence, $Y={\left(Y_{w(1)},\cdots ,Y_{w(n)}\right)}^{T}$, $E={\left(E_{w(1)},\cdots ,E_{w(n)}\right)}^{T}$, $X_{1}={\left(X_{1_{w(1)}},\cdots ,X_{1_{w(n)}}\right)}^{T}$, $X_{2}={\left(X_{2_{w(1)}},\cdots ,X_{2_{w(n)}}\right)}^{T}$, and $\tilde{R}={\left({\tilde{R}}_{w(1)},\cdots ,{\tilde{R}}_{w(n)}\right)}^{T}$.
